# Psychological factors in adherence to COVID-19 public health restrictions in Italy: A path model testing depressed mood, anxiety, and co-rumination via cellphone

**DOI:** 10.1371/journal.pone.0278628

**Published:** 2022-12-02

**Authors:** Michela Balsamo, Karla Klein Murdock, Leonardo Carlucci

**Affiliations:** 1 University of “G. d’Annunzio” Chieti-Pescara, Chieti, Italy; 2 Washington and Lee University, Lexington, Virginia, United States of America; 3 Learning Science Hub, University of Foggia, Foggia, Italy; King Saud University, SAUDI ARABIA

## Abstract

During the COVID-19 pandemic, the success of major non-pharmaceutical interventions, such as quarantine orders, has depended upon robust rates of citizens’ adherence to protocols. Thus, it is critical to public health for research to illuminate factors that affect compliance with contagion-mitigating practices. Previous research has examined sociodemographic factors and aspects of psychological distress as correlates of adherence to public health guidelines. The current study expanded this research to investigate the psychosocial process of co-rumination, which has been identified in previous research as a maladaptive type of social interaction that is associated with elevated levels of anxiety and depression. Data were collected from 932 Italian adults during the initial stages of the highly stressful COVID-19 pandemic and lockdown. A path model was tested to examine multivariate relationships among sociodemographic characteristics, symptoms of psychological distress (i.e., depression and anxiety), co-rumination via cellphone, and self-reported adherence to COVID-19-related public health restrictions. Results revealed that higher rates of co-rumination via cellphone were associated with lower levels of adherence to public health restrictions. Symptoms of depression and anxiety were differentially related to co-rumination processes and adherence to public health restrictions. Higher levels of depression symptoms were directly associated with poorer adherence to public health restrictions, and this path was mediated through higher levels of co-rumination via cellphone. On the contrary, higher levels of state anxiety were directly associated with greater adherence to public health guidelines. This path was also mediated through co-rumination via cellphone. Higher levels of anxiety were correlated with lower levels of co-rumination, which in turn were correlated with lower levels of adherence. These results suggest fruitful directions for future research examining co-rumination as a maladaptive coping behavior that may be addressed within public health interventions.

## Introduction

By 2022 more than 625 million confirmed cases of COVID-19 and 6.5 million deaths have been reported worldwide [1]. Countries across the globe have taken different steps to contain and delay the spread of the virus within their borders, with varying degrees of success. Italian citizens were the first in Europe to experience a COVID-19 outbreak, and their government took swift measures to prevent the disease from spreading, including widespread lockdowns [2–4]. In contrast, the United States waited more than two weeks after the first confirmed case within its borders to enact localized testing procedures [5]. The prolonged prevalence of COVID-19 remains a source of great concern and psychological pressure, both for the general public [6] and within specific sectors of society in particular [7–9].

Compliance with quarantine advice during pandemics has been found to predict other actions that mitigate the risk of contagion [10]. In fact, at the time of recruiting the current sample in Italy during April and May of 2020, the slowing growth in daily reported deaths seemed to evidence a significant impact of the containment and quarantine measures that had been implemented several weeks earlier, according to the Imperial College COVID-19 Response Team [10]. The effective reproduction number, Rt, dropped to close to 1 around the time of lockdown (March 11, 2020). This meant that 38,000 (13,000–84,000) deaths may have been averted.

The success of major non-pharmaceutical interventions depends upon well-organized health services as well as widespread public adherence to protocols. Thus, it is important to identify factors that increase the likelihood that citizens will adhere to quarantine and associated public health orders in Western republics [11–13]. A small body of research, conducted in the early days of the COVID-19 pandemic, has illuminated overlapping sociodemographic and psychological factors that have been associated with adherence to public health restrictions across nations.

### Sociodemographic and psychological correlates of adherence to COVID-19 restrictions

In studies of adults living in Italy, Israel, and the United States, lower adherence to COVID-19-related restrictions has been associated with male gender [14–17], younger age [14, 15, 17], lower levels of education and financial security [15, 18], and residence in nonurban settings [14]. A few studies have investigated associations of psychological distress and adherence to COVID-19 mitigation recommendations during the early days of the pandemic and have produced nuanced results. In Israel, psychological distress, measured with a 10-item scale of anxiety and depression symptoms, was positively associated with non-adherence [16]. In Italy, higher levels of depressive symptoms and lower concerns about becoming infected were associated with lower adherence [18]. In contrast, higher levels of anxiety, as well as higher perceptions of risk and susceptibility regarding COVID-19, were associated with greater adherence to restrictions in Italy [17]. Disparity in the associations of adherence with anxiety versus depression was also identified in an earlier-era meta-analysis of studies investigating associations between depression, anxiety, and compliance with physician-prescribed medical regimens such as treatments, vaccinations, and appointments [19]. These authors found the odds of noncompliance were three times greater for depressed patients when compared to nondepressed patients, but this effect did not extend to patients experiencing anxiety.

Perceptions of COVID-19-related vulnerability and fear may also play a role in adherence to public health guidelines. In a short-term longitudinal study conducted in the United States, feeling personally at risk of COVID-19 infection predicted a greater propensity to engage in hand washing and social distancing behaviors, suggesting that increasing fears about contracting the virus might lead to less risky social behaviors [20]. Similarly, in a study conducted in the United Kingdom, a new measure of COVID-19-related fear was positively correlated with measures of anxiety and depression and with engagement in health-promoting behaviors [21].

Taken together, these results suggest that negative emotions of depression, anxiety, vulnerability, and fear could play complex and different roles in shaping behaviors that are designed to protect public health. Specifically, in spite of the problematic implications of anxiety or fear for psychological well-being, these forms of negative affect may motivate compliance with quarantining, social distancing, and/or hygiene-related behaviors that mitigate the transmission of highly contagious viruses such as COVID-19.

### A potential role of co-rumination in adherence to public health directives

In addition to psychological distress, other psychological processes may affect the likelihood of citizens adhering to health and safety guidelines during public health emergencies such as the COVID-19 pandemic. For instance, co-rumination refers to a tendency to engage in extensive, negatively focused discussions in which one’s reactions to ongoing problems are repeated and rehashed in a dyadic context [22]. This construct is conceptualized as an interpersonal form of rumination that unites the content of cognitive rumination (i.e., perseverative thoughts about problems and/or symptoms) [23] with self-disclosures in social interactions [22].

Co-rumination has been associated with socioemotional costs and benefits [24]. Co-rumination has been consistently associated with increased emotional intimacy and positive perceptions of friendship quality in youths [25], and also with increased anxiety and depressive symptoms in cross-sectional and prospective studies [25–31]. Co-rumination has been found to predict increases in levels of internalizing symptoms [27], which in turn contribute to subsequent increases in co-rumination [27, 32]. Longitudinal studies on co-rumination and psychological distress support both directions of effect [32, 33].

Literature on co-rumination has begun to illuminate contexts and conditions that drive and/or exacerbate its association with psychosocial distress. The association between co-rumination and depression is amplified under conditions of high stress [26, 34]. In particular, it appears that exposure to interpersonal stress can shape the implications of co-ruminative behavior for mental health [30, 32, 35]. Co-rumination about interpersonal stressors, but not other types of stressors, has been associated with depression [36].

As suggested by Starr and colleagues, the COVID-19 pandemic may represent a unique climate that gives rise to co-rumination [37]. For many people, the pandemic has led to shifts in the quality and availability of physical and emotional support within social networks [37]. Thus, social distancing, lockdowns, school closures, academic disruptions, physical separation from family and friends, educating and caring for children at home, self-quarantine following virus exposures, and other similar COVID-19-related events and policies could be conceptualized as pandemic-related interpersonal stressors. In addition, sheer uncertainty associated with the pandemic, a novel virus with an unknown long-term sequelae and treatment and massive economic fallout, may contribute to excessive focus on these problems and associated distress when support-seeking. These stressors could increase symptoms of depression and/or anxiety as well as the tendency to engage in co-ruminative discussions. Research has shown that unusually high rates of internalizing symptoms have been endorsed during the COVID-19 era [14], and this may increase the likelihood that both support seekers and support providers will engage in co-rumination due to emotional strain.

Three studies have investigated co-rumination within the context of the pandemic. Co-rumination about COVID-19 stressors was longitudinally associated with symptoms of anxiety and depression in the United States between April and May of 2020 [38]. Similarly, Stone and Veksler [39] found that co-rumination about COVID-19 was associated with higher levels of depression, anxiety, and health-related anxiety among adults in the United States between March and May of 2020. Finally, a recent cross-sectional study found evidence for links among COVID-19-focused co-rumination, internalizing symptoms, and “committed action”, which refers to engaging in behaviors that are consistent with one’s values even in the context of challenges and setbacks [37, 40]. These authors found that COVID-19-focused co-rumination correlated positively with internalizing symptoms and negatively with general forms of value-driven committed action. In particular, the passive co-brooding component of co-rumination moderated the association of COVID-19-related stress with both internalizing symptoms and committed action [37]. Higher levels of COVID-19-related stress were associated with higher levels of internalizing symptoms only at higher levels of co-brooding co-rumination.

Taken together, these studies suggest that co-rumination is a pattern of behavior that may have important implications for citizens’ psychological well-being as well as for their compliance with public health measures that keep them and their communities healthy and safe.

### The current study

This study examined multivariate relationships among sociodemographic characteristics, anxiety and depression symptoms, co-rumination, and self-reported adherence to national health guidelines and restrictions during the highly stressful early days of the COVID-19 pandemic in Italy. A path model was tested to examine direct and indirect relationships of anxiety, depression, and co-rumination with adherence. In this study we examined cellphone-mediated co-ruminative behaviors, as participants were quarantined with severely restricted access to face-to-face contact with others. In previous research, co-rumination via cellphone has been associated with compromises in psychosocial well-being within contexts of interpersonal stress [35].

Increases in psychiatric symptoms are often expected during a large-scale disaster, and in fact elevated levels of anxiety, psychological distress, insomnia, posttraumatic stress disorder, and depression have been found during the COVID-19 pandemic [41–43]. Thus, our hypothetical model tested a direction of effects running from anxiety and depression through co-rumination to predict variability in adherence. Given the inconsistent findings in investigations of various psychological distress indicators with adherence [16, 19], we tested the separate effects of anxiety and depression on adherence on an exploratory basis.

The lockdown phases of Italy’s initial response to the COVID-19 pandemic involved a high degree of potential interpersonal stress associated with isolation, social distancing, and the disruption of social routines. Social support seeking is commonly conceived as an adaptive way for alleviating distress and promoting wellbeing [44]. Although co-ruminative interactions may involve aspects of social support seeking such as (“*disclosing about problems [which] conveys trust and allows the friend to offer support*” p.177) [45], these potential benefits may be outweighed by the costs of this coping behavior. Previous literature has identified co-rumination as a maladaptive type of coping, particularly during times of high stress [26, 34] and in response to interpersonal stressors [36]. Furthermore, recent findings have highlighted the maladaptive and ineffective role of co-rumination in responses to COVID-19-related stressors [37, 46]. Thus, we predicted that co-rumination would be negatively related to compliance with public health restrictions. We expected that co-rumination would serially mediate the relationship between depression and anxiety symptoms and adherence to national public health guidelines in a possible causal path.

## Method

### Procedure

Data for this study were collected between April 24 and May 25^th^, 2020, during the COVID-19 lockdown imposed by the Italian government (Phase 2) [47]. Italy was the first country in the world to implement a nationwide quarantine in response to COVID-19, and at the point of recruitment for the current study, Italian citizens had been quarantined in their homes, with travel restricted and non-essential businesses and schools closed, for 6–8 weeks.

Participants included quarantined Italian adults, aged 18 years and older, with access to a networked computer. Participants completed an online web-based survey programmed via QUALTRICS® and accessed through mail and social media using a virtual snowball sampling method [17].

### Ethic statements

The study was recorded to the Ministry of Education, University and Research (MIUR) and approved by the Ethical Review Board at the Department of Psychological Sciences, Health and Territory, University of Chieti, Italy, see also [17, 18]. All participants signed an electronic informed consent that included information on the purpose of the study, the methods, the advantages of participation, the voluntary involvement, and the researchers’ contact information.

### Sample

From a pool of 1961 individuals who accessed the survey, 1029 respondents were excluded because they failed more than one validity check item, did not complete the survey, or failed to complete >50% of survey questions. The final sample included 932 participants.

Of the 932 respondents included in the analyses, 660 self-identified as female (70.8%) and 272 self-identified as male (29.2%). The mean (±SD) age of the participants was 29.29±11.02 years. Among these, 479 (51.4%) respondents held a high school diploma, while 379 (40.7%) held a higher degree (bachelor/master/doctorate). Concerning marital status, 635 (68.1%) participants were unmarried/single, 173 (18.6%) were married, 16 (1.8%) were divorced/separated, and 105 (11.3%) were cohabiting. The majority of participants were located in the South of Italy (i.e., 14,8% Campania; 11.8% Abruzzo; 10,3% Apulia regions), and one quarter of the sample was comprised of undergraduate students (25.4%). About one third of respondents (33.5%) were employed and their average yearly income ranged from 10,000–30,000 €. Respondents reported living during the past week in quarantine with family (59.8%), with colleagues/roommate/other familiars (11.3%), or alone.

### Measures

Respondents provided information regarding their sex, age, education, marital status, geographic region of residence, employment status, and annual income.

### Depression

The 21-item Teate Depression Inventory (TDI) [48–50], developed via Rasch analysis, was employed to evaluate depressive symptoms in the past two weeks. Responses were made on a 5-point Likert scale ranging from 1 “*never*” to 5 “*always*”. Total scores were created by first reverse-coding several items (e.g., “*I felt as though I had enough energy to perform my daily activities*”), and then averaging items. Higher TDI scores reflect higher levels of depression. Cronbach’s α coefficient in our study was .93.

### State anxiety

The 21-item state scale of the State-Trait Inventory for Cognitive and Somatic Anxiety (STICSA-S) [51–53] was administered to evaluate cognitive (e.g., “*I have trouble remembering things*”) and somatic (e.g., “*My muscles are tense*”) symptoms of state anxiety. Individuals rated how often a statement was true for them in the past two weeks, from 1 “*not at all*” to 4 “*very much so*”. A total score of anxiety was created by averaging items, with higher scores reflecting higher levels of state anxiety. Cronbach’s α coefficient was .90.

### Co-rumination via cellphone

The 27-item co-rumination questionnaire (CRQ) [22] was translated to Italian [22, 54, 55] and modified to assess participant’s tendency to excessively discuss problems in cellphone-mediated interactions within close relationships [56]. Participants were asked to think about the person with whom they had communicated the most on their smartphone or cellphone through talking or videoconferencing apps (e.g., FaceTime, Zoom, Google hangout) during the past week. They initially responded to the open-ended question: “*What kind of problems or stressors have you usually communicated about*? *(Please be as specific as possible*.)” Next, they responded to items regarding the frequency and nature of co-ruminative interactions (e.g., “*After this person told me about a problem*, *I always tried to get them to communicate more about it later*”; “*When we communicated about a problem that one of us had*, *we tried to figure out every one of the bad things that might happen because of the problem*.”) Participants responded on a 5-point Likert scale ranging from 1 “*not at all true*” to 5 “*really true*”. Total scores for co-rumination via cellphone were created by averaging the 27 items, with higher scores reflecting higher levels of co-rumination via cellphone. Internal consistency was excellent in this sample (α = .95).

### Adherence to COVID-19 public health guidelines

Participants reported their compliance during the past week with behavioral guidelines recommended or required to prevent the spread of the COVID-19 virus [17]. Eleven items assessed preventive (e.g., handwashing) and avoidant (e.g., social contact) behaviors and participants responded on a 5-point likert scale ranging from 1 “*never*” to 5 “*always*”. A global adherence index was calculated by averaging the items, with higher scores reflecting greater adherence. In the present sample, Cronbach’s α coefficient was .66.

### Data analysis

Descriptive statistics (i.e., means, standard deviations, and indices of skewness and kurtosis), were computed to assess departures from normality. Cronbach’s alphas were also computed to test the internal consistency of primary study variables. Assumptions of path analysis (e.g., residuals, missing data, and collinearity) were checked and addressed before conducting primary analyses. Path analysis with observed variables was used to determine the pathways by which socio-demographic variables, anxiety, depression, and co-rumination influenced adherence to quarantine guidelines. Path analysis yields indices of direct and indirect effects between the variables as well as the overall suitability of the model.

As suggested in previous studies [17, 18], in the first model exploratory analyses were conducted in which socio-demographic variables (age, sex, income per year, education) were regressed on anxiety, depression, and adherence, as well as on co-rumination via cellphone [55, 57]. At the same time, co-rumination via cellphone was proposed in the model to mediate both the depression-adherence (mediation #1), and anxiety-adherence (mediation #2) relationships, respectively. Both anxiety and depression symptoms were allowed to have direct and indirect effects on adherence to public health guidelines. Due to the high overlap, the covariance error between anxiety and depression was set free to estimate.

To determine the adequacy of the model, the chi-square test fit value was examined taking into consideration the sample size. As the significance of a chi-square test is dependent on the number of participants, other goodness-of-fit indexes were also used, including the Root Mean Square Error of Approximation (RMSEA) and its 90% Confidence Interval (90% CI); the Non-Normed Fit Index (NNFI); the Comparative Fit Index (CFI); and the Standardized Root Mean Square Residuals (SRMR). In the present study, NNFI and CFI values of .95 and above were considered to reflect adequate fit and values of .97 and above were considered to indicate an excellent fit. RMSEA and SRMR values of .08 and .10 or less were considered to reflect an adequate fit and values of .05 or less were considered to reflect a good fit [58].

Based on modification indices tests, post-hoc modifications were made to the proposed model to remove or add new paths as necessary. The statistical significance of all direct and indirect effects was evaluated to determine which variables had a direct and indirect impact on adherence. Standardized beta coefficients (β) were derived for each explanatory variable to allow for the comparison and estimation of the relative importance of each measure. The *R*^2^ value was calculated for the outcome variable to determine the proportion of variance explained by the model. Mediation analyses were evaluated by analysing the significance of the indirect effect between predictors and adjustment components by bootstrapping. Data were analysed using the IBM SPSS software (version 24.0), semPower R-package [59], and Mplus 7 [60]. A *p* < .05 was employed as the threshold for statistical significance.

## Results

Data were cleaned and screened for out-of range values, univariate and multivariate normality, and normality. Means, standard deviations, and ranges of the variables included in the model were examined and are presented in Table 1. In the open-ended assessment of co-rumination via cellphone content, 99% of participants reported that they had co-ruminated about COVID-19 and its consequences during the past week.

**Table 1:** Descriptive statistics of the variables included in the hypothesized model.

	Descriptive					
	M	SD	Range	Skewness	Kurtosis	α
TDI	1.676	.734	0–4	.203	-.402	.93
STICSA_S	1.694	.507		.817	.236	.90
CRvC	2.824	.865		.102	-.715	.95
ADHER	4.075	.441		-.796	1.439	.66

*Note*. TDI = Teate Depression Inventory; STICSA_S = State-Trait Inventory for Cognitive and Somatic Anxiety–State scale

CRvC = Co-rumination via cellphone; ADHER = Adherence to COVID-19 public health guidelines, α = Cronbach’s alpha.

No univariate outliers nor multivariate outliers were detected as assessed by standardized *z*-scores and Mahalanobis Distance test (below the cut-off χ^2^ = 18.47, *p*< .001). All primary study variables were deemed to be normally distributed, since they did not exceeded a kurtosis value > |7| and a skewness value > |2| [61]. Residuals errors were approximately normally distributed, and the variance inflation factor (VIF) values (range 1.05–2.08) confirmed that collinearity assumption were met. Correlations among the primary study variables were also examined. All the variables included in the model were significantly correlated with the adherence outcome variable, and inter-correlated with values of *r*_*P*earson_ ranging from .11 to .31. As expected, depression as measured by the TDI was moderately to highly correlated with the STICSA-state scale (*r* = .70).

### Model fit evaluation

The model was tested using a multivariate statistical model with Maximum Likelihood (ML) estimation, assuming multivariate normality. According to the post-hoc power analysis (df = 10, α = .05, and RMSEA = .05), our sample size ensured an adequate statistical power (1-β = 94%) in order to obtain unbiased estimates and standard errors.

**Fig 1:**
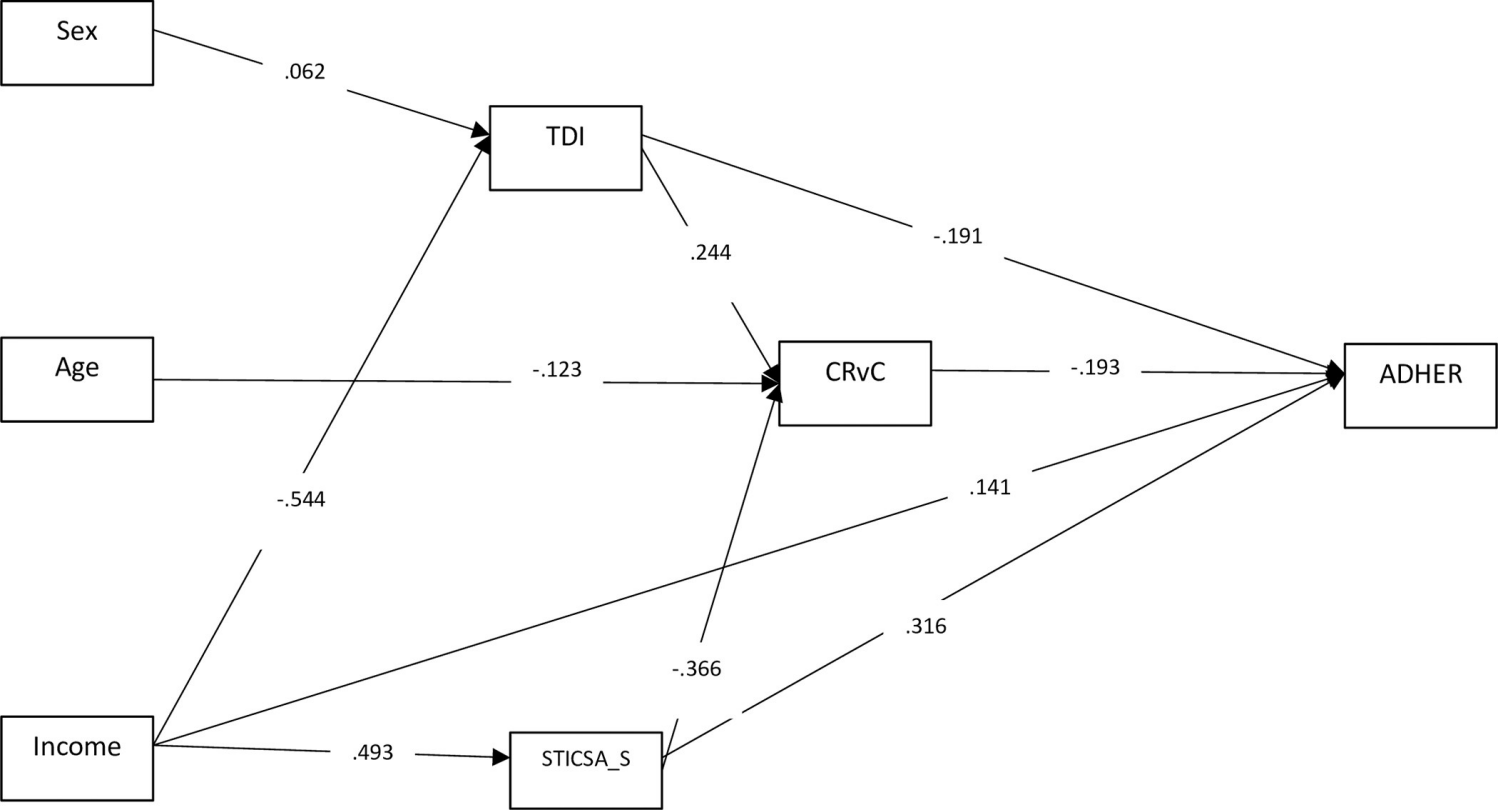
Path analysis with standardized direct effects. CI_95%_ = bias-corrected bootstrap confidence interval at 95% (10000 samples). *Note*. TDI = Teate Depression Inventory; STICSA_S = State-Trait Inventory for Cognitive and Somatic Anxiety–State scale; CRvC = Co-rumination via cellphone; ADHER = Adherence to COVID-19 public health guideline.

Our baseline model did not present a satisfactory global adjustment for our data, with fit indices of χ2(8) = 73.14 (*p*< .001); CFI = .962; TLI = 875; RMSEA = .093 (90% CI = (.075; .114)); and SRMR = .06. In order to improve the model, the non-significant path/variables were deleted from the model in turn, and a new model was tested iteratively. On the basis of the modification indices, both marital status and education variables were entirely discarded from the final model because they did not display significant associations with the outcome. The refined model showed a good fit global adjustment with χ2(8) = 45.88 (*p*< .001); CFI = .979; TLI = .963; RMSEA = .062 (90% CI = (.045; .081)); and SRMR = .56. The *R*^2^ indicated that this model (Fig 1) explains 45% of the variance in adherence, 31% of the variance in co-rumination, 24% in state anxiety, and 30% in depression.

In the refined model (see Fig 1), among socio-demographic variables, sex had a direct effect on depression (β = -.062; *p*< .01) and age had a direct effect on co-rumination via cellphone (β = −.123, *p*< .001). Income had direct effects on depression (β = -.544; *p*< .001), anxiety (β = .493; *p*< .001), and adherence (β = .141; *p*< .01). Both state anxiety (β = .244, *p*< .001) and depression (β = −.366, *p*< .001) had a direct effect on co-rumination, but in opposite directions. Depression (β = −.191, *p*< .001), anxiety (β = .316, *p*< .001) and co-rumination via cellphone (β = −.193, *p*< .001) had a direct effect on adherence.

Next, in order to test hypotheses about individual parameters, and to test the equality of parameters, we performed the Wald test. As expected, all the direct effects observed on adherence (via income, depression, anxiety, and co-rumination via cellphone) were statistically different (*p* < .001) from one another, except for the difference among depression/adherence vs. co-rumination/adherence paths (W = .167(1), *p* < .001).

Depression had an indirect effect on adherence through the mediator of co-rumination via cellphone (β = -.047; *p*< .001; CI -.061 –-.035). Likewise, anxiety had an indirect effect on adherence through the mediator of co-rumination via cellphone (β = .071; *p*< .001; CI .058 –.085). A third set of mediational paths was identified by the model that showed income as a predictor of adherence throughout the mediators of anxiety and depression. As displayed in Table 2, income had an indirect effect in adherence, mediated by anxiety (β = .156; *p*< .001; CI .117 –.127) as well as by depression (β = .104; *p*< .001; CI .083 –.127). With a 95% confidence interval which did not include zero, all the indirect paths noted above were significantly greater than zero at α = .05, confirming the mediation effects. Other non-mediational indirect effects are displayed in Table 2.

**Table 2:** Standardized indirect effects for mediation and other indirect effects.

*Variables*			Indirect Effect	CI_95%_	
Predictor	Mediator	Outcome		Lower	Upper
TDI	CRvC	ADHER	-.047**	-.061	-.035
STICSA_S	CRvC	ADHER	.071**	.058	.085
Income	STICSA_S	ADHER	.156**	.117	.200
Income	TDI	ADHER	.104**	.083	.127
*Other indirect effects (no mediation)*					
Predictor	*via *	Outcome			
Sex	TDI	ADHER	-.012*	-.020	-.005
Sex	TDI × CRvC	ADHER	-.003*	-.005	-.001
Age	CRvC	ADHER	.024**	.014	.034
Income	STICSA_S × CRvC	ADHER	.035**	.028	.043
Income	TDI × CRvC	ADHER	.026**	.019	.033

** *p*< .001

* *p*< .05

CI95% = Bootstrap bias-corrected confidence interval at 95% (1000 samples)

*Note*. TDI = Teate Depression Inventory; STICSA_S = State-Trait Inventory for Cognitive and Somatic Anxiety–State scale; CRvC = Co-rumination via cellphone; ADHER = Adherence to COVID-19 public health guideline.

## Discussion

During public health crises, it is crucial to understand risk factors and processes that may exacerbate negative health outcomes. In response to the COVID-19 pandemic, the Italian government mandated several weeks of strict quarantine and recommended a host of behaviors aimed at reducing virus transmission. The current study tested a hypothetical model framing co-rumination via cellphone as a mediator between specific aspects of psychological distress (i.e., anxiety and depression) and self-reported adherence to public health guidelines in Italy during the early months of the pandemic.

Nearly all participants in our sample endorsed co-ruminating about COVID-19-related stressors in cellphone-mediated interactions during the late spring of 2020. Previous research has demonstrated that cellphones are used as vehicles for co-rumination [35, 56], and it is not surprising that these interactions focused on COVID-19 during a time that face-to-face interactions were severly restricted, daily activities were suppressed, and media sources were saturated with COVID-19-related content.

As expected, in the current study higher levels of co-rumination were associated with poorer adherence to public health guidance. This is consistent with previous research demonstrating that co-rumination intensified the association between stress and symptoms [37]. As predicted, in the present cross-sectional study we found that co-rumination serially mediated the relationship between internalizing symptoms and committed actions regarding personal and public health.

The results of this study reinforce the importance of examining unique effects of anxiety and depression symptoms, as opposed to global measures of psychological distress, on adherence. Consistent with previous studies specifically focusing on depression, in this study higher rates of depression were directly associated with higher levels of co-rumination [28, 39] and lower rates of adherence [19]. In the current study, the relationship between depression and adherence was mediated through co-rumination via cellphone; higher levels of depression were associated with higher rates of co-rumination, which in turn were associated with poorer adherence. It should be noted that despite studies suggesting that the association between co-rumination and depression is amplified under conditions of high stress [26, 34], our correlation coefficients and beta coefficients were just slightly above the averages (*r*s range .14-.26) identified in Spendelow, Simonds [29] meta-analytic study.

In contrast to our findings for depression, in this study higher rates of anxiety were directly associated with higher rates of adherence, perhaps reflecting a potentially adaptive role of anxiety [17] and/or perceptions of vulnerability [20] with respect to health-promoting behaviors during the pandemic. The relationship between anxiety and adherence was mediated through co-rumination via cellphone; higher levels of anxiety were associated with lower rates of co-rumination, which in turn were associated with poorer adherence.

Given that previous research has found positive relationships between anxiety and co-rumination [39, 55, 62], future research is necessary to clarify why, in the current study, higher rates of anxiety were associated with lower levels of co-rumination. One possibility is that this effect emerged from specific qualities of the anxiety participants experienced during the early months of the pandemic. For instance, it is possible that when compared to generalized or trait anxieties, forms of state anxiety arising from the pandemic (e.g., health vulnerability, financial insecurity, social isolation, interpersonal stressors, or other aspects of routine disruption), or the cumulative effect of these anxieties, made it less likely that individuals would engage in cellphone-mediated co-ruminative interactions.

The present finding of an inverse relationship between anxiety and co-rumination also may be clarified in future research by investigating subcomponents of co-rumination such as brooding, which describes a passive tendency to dwell on and catastrophize problems and reflection, which involves a more active tendency to make causal analyses and gain insight. Previous research has shown that of these components, brooding is the more maladaptive ruminative process that is responsible for negative outcomes [63–65]. As previously noted, in the recent study by Starr, Huang [37], co-brooding and co-reflection each moderated the association between COVID-19 stress exposure and a) internalizing symptoms (i.e., COVID-19-related fears and depressive symptoms) and b) committed actions, but in opposite directions, with co-brooding predicting increased symptoms and decreased committed action, and co-reflection predicting the opposite. When individuals engage in passive brooding in dyadic interactions, it may by nature prevent their engagement in active problem solving and instead serve as a way to avoid directly confronting the stressors in their lives.

### Socio-demographic correlates

In this sample, income was found to be directly and indirectly (via depression and state anxiety) associated with adherence. In line with the recent findings, participants’ self-reported income was positively correlated with their compliance with public health recommendations [66, 67]. Given the central role of social distancing in these recommendations [67], it may be conceptualized as a “privilege” dependent on financial resources. People with higher income tend to reside in less-crowded living situations, making it easier for them to comply with social distancing [68]. On the contrary, people with lower incomes may be more likely to live in crowded housing situations and/or less able to work from home and self-isolate when required [69, 70]. Besides, in our study people with lower income were found more depressed than anxious. This datum was in line with other studies that showed a predominance of moderate-to-severe symptoms across both disorders in samples of young adults and university sample from urban, low-income public university [71]; and partially agree to other preliminary population-level health assessments conducted during COVID-19 pandemic [72].

A possible explanation about the negative relationship among depression and income in to predict adherence to quarantine guidelines is that the COVID-19 pandemic may be exacerbating a situation of hopelessness and stigmatization among youth as they have the feeling that their future is unknow [41].

Despite past studies indicating that the socio-demographic factors of age, sex, education, marital status, and employment status are correlated with adherence to preventative behaviours against respiratory illnesses [73–75], none of these variables were directly related to the adherence to COVID-19 quarantine guideline in the current study. However, age was found to indirectly predict adherence in that older participants reported less co-rumination via cellphone, which in turn was related to higher levels of adherence. It is well established that individuals’ tendency to co-ruminate varies considerably, and a maladaptive effect of co-rumination in adults has been related to the interaction between age and sex variables [76, 77]. The few studies that have taken into account concerns about COVID-19 and depression showed that older adults were among the main facilitators for following public health guidelines, particularly with respect to physical distancing directives [78, 79]. Furthermore, in a study by Guan and colleagues, estimates of effect in compliance to quarantine guidelines increased when their model was adjusted for more than one demographic variable (such as age, sex, ethnicity, income and education) [79].

Taking together, our results extend the current literature on compliance to protective measures that is largely unaffected by age, sex and educational level [17, 80–82]. Further research is warranted to clarify underlying causal mechanisms and to shed light onto the interaction effects of socio-demographic variables on adherence.

### Limitations

Although the current study has the strength of testing a multidimensional model addressing the role of co-rumination in citizens’ compliance with behavioral COVID-19 mitigation guidelines, its methodology is characterized by several limitations. First, we used a global measure of co-rumination via cellphone, which does not provide information about face-to-face co-rumination or about separate co-brooding and co-reflection components [34, 65]. Differentiating between in-person and cellphone-mediated co-rumination and measuring nuances of co-ruminative interactions could add explanatory power to the model. However, it should be noted that the Co-Rumination Questionnaire [22] was not originally developed to differentiate between these two constructs. Indeed, factorial studies on the CRQ were consistent with one strong factor solution, and a two factor solution was not the same across studies; it was retained just for conceptual reasons [45]. Second, we used an ad hoc measure of adherence to COVID-19 public health guideline. This index has been developed during the isolation phase (lockdown) according to the Italian laws and guidelines [17], thus its use is limited to the Italian context. Future studies are required to assess its validity and generalizability to other indexes of compliance with public health measures. Studies in this way may account for the geographically widespread and meaningful changes in adherence behaviours as observed worldwidely [83].

Third, our path model imposed a predictive relationship between internalizing symptoms and co-rumination, and tested co-rumination as a mediator through which these symptoms may affect adherence. Although this proposed direction of effects is in line with other studies [84], several cross-sectional studies have tested models in which co-rumination was proposed to predict increases in internalizing symptoms, as well as in friendship quality and closeness [55, 57]. As suggested by Rose [22, 45], experiencing internalizing symptoms and having a close friend also could contribute to increased co-rumination. As noted above, results of longitudinal studies have supported both directions of effects between co-rumination and internalizing symptoms [28, 32, 33, 85]. It is important for future longitudinal studies to investigate nuances in the nature of transactional effects among anxiety, depression, co-rumination, and compliance with health-promoting behaviors. Future longitudinal study should also include as possible representative samples to guarantee generalizability and estimation accuracy of compliant behaviours. Our sample has mainly composed by young and female participants from the south Italy. This could in part explained by the fact that co-rumination is a age-related phenomena [45], and females are more compliant and maintained adherence over time [83].

Finally, it is important to note that levels of COVID-19-related stress and uncertainty, as well as restrictiveness of public health guidelines, have changed over time. The current study was conducted during Italy’s Phase 2 lockdown period and thus it assessed a particular time and context in which uncertainty was high and internalizing symptoms may have been particularly pronounced.

## Conclusions

During public health crises, it is crucial to understand cognitive and behavioral factors that affect citizens’ compliance with best practices for health and safety. During the initial months of the COVID-19 pandemic, when participants of this study were responding to lockdown conditions in Italy, the nature of their psychological distress (i.e., anxiety versus depressed mood) was significantly associated with their propensity to co-ruminate about stressors and problems, including COVID-19, in their interactions with others. Depressed mood was positively associated with co-rumination and inversely associated with adherence to public health restrictions. In contrast, state anxiety was inversely associated with co-rumination and positively related to adherence. Future research unpacking the nature of these differential relationships may shed light on the function of co-rumination as a coping behavior and predictor of public health compliance during times of widespread uncertainty and stress.

## Supporting information

S1 Data (CSV)
